# A combination of proviral and antiviral roles of CD11c- and T-bet-expressing B cells defines parameters of chronic murine gammaherpesvirus infection

**DOI:** 10.1128/mbio.02992-25

**Published:** 2026-01-30

**Authors:** Erika R. Johansen, Xander G. Bradeen, Emily V. Xie, Bonnie N. Dittel, Elizabeth A. Leadbetter, Vera L. Tarakanova

**Affiliations:** 1 Department of Microbiology and Immunology, Medical College of Wisconsin 735651, Milwaukee, Wisconsin, USA; 2 Medical Scientist Training Program, Medical College of Wisconsin 5506, Milwaukee, Wisconsin, USA; 3 Versiti Blood Research Institute 418550 , Milwaukee, Wisconsin, USA; 4 Department of Microbiology, Immunology & Molecular Genetics, UT Health San Antonio Long School of Medicine 557944 , San Antonio, Texas, USA; 5 Cancer Center, Medical College of Wisconsin 166013, Milwaukee, Wisconsin, USA; The University of North Carolina at Chapel Hill, Chapel Hill, North Carolina, USA

**Keywords:** gammaherpesvirus, age-associated B cells, germinal center response, chronic infection, latent reservoir, self-reactive B cells, T-bet-expressing B cells

## Abstract

Gammaherpesviruses are ubiquitous pathogens that establish lifelong infection and are associated with the development of cancer and multiple sclerosis. Unlike other viral families, gammaherpesviruses selectively target B cells to establish chronic infection. Specifically, gammaherpesvirus-driven differentiation of latently infected cells through the germinal center supports chronic infection and seeds viral lymphomagenesis. CD11c^+^ B cells are induced by most viral infections and are also observed in aged individuals and autoimmune diseases. Classically, CD11c^+^ B cells differentiate via an extrafollicular pathway that does not involve germinal center response, generating antibodies of beneficial (antiviral) or pathogenic (self-reactive) nature. While CD11c^+^ B cells are induced during B cell-tropic gammaherpesvirus infection, their role in chronic infection remains poorly defined. Here, we demonstrate that infection of the CD11c^+^ B cells, including those expressing germinal center markers, contributes to the overall latent gammaherpesvirus reservoir during natural infection. Both T-bet^+^ and T-bet^neg^ CD11c^+^ B cell subsets expanded and underwent germinal center differentiation during chronic gammaherpesvirus infection. Furthermore, B cell-intrinsic T-bet expression attenuated the long-term latent viral reservoir, gammaherpesvirus-driven germinal center responses, and differentiation of self-reactive B cells. In summary, our study for the first time defines CD11c^+^ splenic B cells as a reservoir of latent gammaherpesvirus during mucosal chronic infection and reveals an important role of T-bet^+^ B cells in controlling long-term infection and gammaherpesvirus-driven pathogenic host processes.

**IMPORTANCE:**

Gammaherpesviruses are ubiquitous pathogens that are associated with cancer and multiple sclerosis. These viruses selectively infect B cells and drive their differentiation through the germinal center response to establish chronic infection. Here, we demonstrate that gammaherpesvirus infection drives expansion and germinal center-based differentiation of CD11c^+^ B cells that host the latent viral reservoir. We also show that B-cell-intrinsic T-bet expression is important for control of long-term gammaherpesvirus infection and pathogenesis.

## INTRODUCTION

Gammaherpesviruses, such as Epstein-Barr virus (EBV) and Kaposi’s Sarcoma-associated herpesvirus (KSHV), are highly prevalent pathogens that establish lifelong infection in >90% of adults worldwide and are associated with the development of cancer and multiple sclerosis (1–4). Unlike other virus families, gammaherpesviruses target B cells to establish lifelong infection (5–10). Specifically, gammaherpesviruses infect naïve B cells and drive both infected and uninfected counterparts into the germinal center, where robust proliferation of infected B cells leads to an exponential increase in the splenic latent reservoir (8, 10, 11). The subsequent differentiation of latently infected germinal center B cells into memory B cells or antibody-secreting plasma cells provides reservoirs for lifelong infection and viral reactivation, respectively (12, 13). In contrast to other viral infections, gammaherpesviruses selectively drive the differentiation of self-reactive B cells with the long-term latent infection established in memory B cells that encode a self-reactive B-cell receptor (14). Aside from its role in supporting the expansion of the splenic latent reservoir, the germinal center stage of B-cell differentiation is believed to be a target of gammaherpesvirus-driven lymphomagenesis, as many EBV^+^ lymphomas are of germinal center or post-germinal center origin (15). Although the risk factors of gammaherpesvirus disease cannot be precisely defined at an individual level, poor control of chronic infection, as evidenced by an increased viral latent reservoir and viral reactivation, precedes the development of gammaherpesvirus-associated cancers (15–21).

The current study defines the interaction of a murine B cell-tropic gammaherpesvirus with age-associated B cells (ABCs), also known as atypical memory B cells (22). ABCs represent a highly heterogeneous B cell population and have been classically defined by expression of CD11c, an integrin expressed predominantly on myeloid cells, and T-bet, a transcription factor that is involved in differentiation of T cells. Recently, the requirement of T-bet as a defining marker of ABCs has been called into question by studies establishing that T-bet^neg^ ABCs have similar characteristics and functions as their T-bet^+^ counterparts (23). The role of CD11c expression in the biology of ABCs remains unclear due to the lack of experimental approaches that specifically target CD11c^+^ B cells. CD11c^+^ B cells expand during aging; however, they also increase in viral infections (24–27), immunizations (28, 29), and autoimmune diseases (30, 31). CD11c^+^ B cells that expand during autoimmune diseases can be pathogenic and produce self-reactive antibodies (32). In contrast, T-bet^+^ B cells induced by influenza infection are protective via the generation of antiviral antibodies (25).

The role of CD11c^+^ B cells in the context of EBV and KSHV infections has not been defined, in part due to the challenge presented by the species specificity of human gammaherpesviruses. To overcome that challenge, the current study uses murine gammaherpesvirus 68 (MHV68), a natural rodent gammaherpesvirus that provides a powerful model of chronic gammaherpesvirus infection of an intact host. Importantly, MHV68 shares key features with EBV, including a high degree of genetic conservation (33–35), hijacking of B-cell germinal center differentiation processes (5, 36) and B-cell lymphomagenesis (15, 37).

Two recently published studies from the Horwitz group have explored the interaction between MHV68 infection and CD11c^+^ B cells, including in the context of experimental autoimmune encephalomyelitis and using a mouse model of B cell-specific T-bet deficiency (38, 39). ABC analyses in the two published studies were limited to class-switched, IgD^neg^T-bet^+^CD11c^+^ B cells and exclusively relied on intraperitoneal inoculation of MHV68. While the intraperitoneal route of inoculation may mimic iatrogenic gammaherpesvirus infection (i.e., EBV seronegative recipient of an organ transplant from EBV seropositive donor), natural gammaherpesvirus infection usually occurs following mucosal viral transmission. Correspondingly, many viral and host phenotypes of chronic MHV68 infection are profoundly altered by inoculation route (40–44). A single foundational publication in the ABC field (27) used a broader ABC immunophenotyping to demonstrate expansion of T-bet^+^CD11c^+^ splenic B cells following mucosal MHV68 inoculation. In this study, sorted CD11c^+^ B cells could produce anti-MHV68 antibodies *ex vivo*. Furthermore, using a mixed bone marrow chimera approach, the published study demonstrated increased MHV68 genome copy number at 18 days post-infection of recipients lacking T-bet expression in B cells. However, the extent to which CD11c^+^ B cells host the latent viral reservoir or support MHV68-driven increase in self-reactive antibody titers remains unknown.

In this study, we found that the gammaherpesvirus latent reservoir was equally supported by CD11c^+^ and CD11c^neg^ splenic B cells during the establishment of chronic infection, defining the CD11c^+^ B-cell population as a host of gammaherpesvirus latency. Both T-bet^+^ and T-bet^neg^ CD11c^+^ B-cell subsets expanded during chronic infection and expressed germinal center markers. B cell-specific depletion of T-bet led to decreased latent infection of CD11c^+^ germinal center B cells. Long-term infected mice with a B cell-specific T-bet deficiency demonstrated an increase in the splenic latent reservoir, germinal center B cells, and titers of self-reactive, but not virus-specific IgG. These findings suggest that T-bet^+^ B cells contribute to long-term control of chronic gammaherpesvirus infection and virus-driven pathogenic differentiation of self-reactive B cells.

## RESULTS

### MHV68-driven germinal center differentiation of T-bet^+^ and T-bet^neg^ CD11c^+^ splenic B cells supports the latent viral reservoir

**Fig 1:**
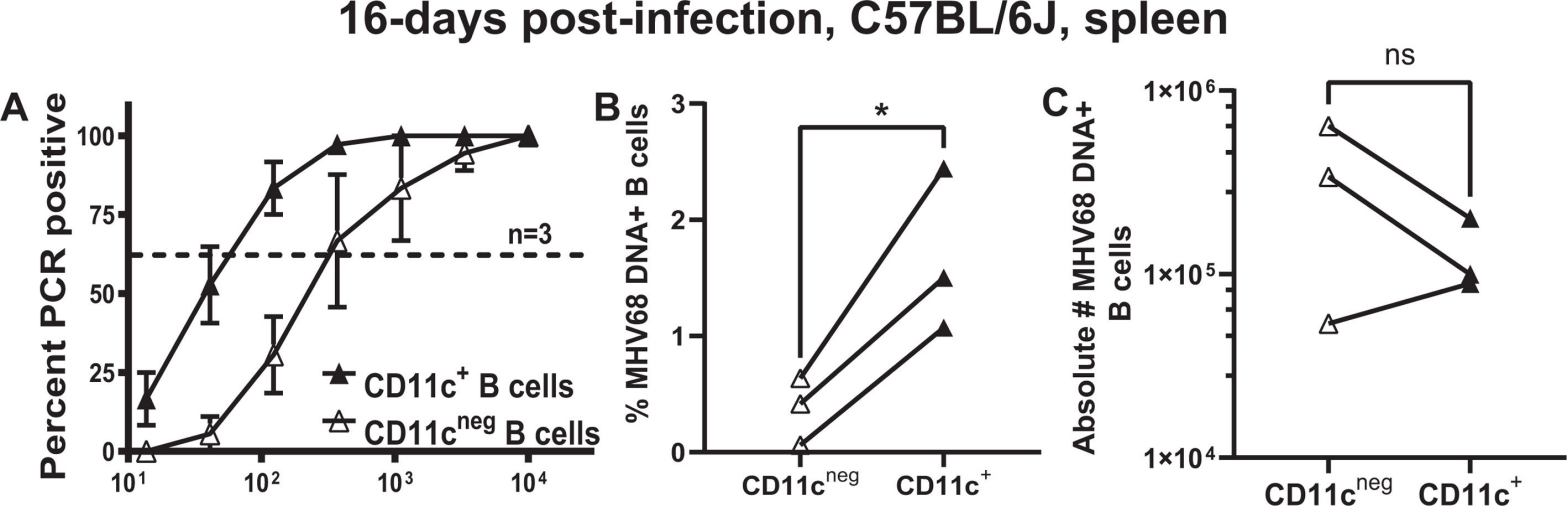
Latent MHV68 reservoir is enriched in splenic CD11c^+^ B cells. C57BL/6(J) mice were intranasally infected with 10,000 PFU of MHV68, and splenocytes were analyzed at 16 days post-infection. Pooled splenocytes from infected animals were enriched for CD19^+^ B cells and further separated into B220^+^CD19^+^CD11c^+^ and B220^+^CD19^+^CD11c^neg^ populations by fluorescence-activated cell sorting (FACS). The frequency (**A**) of MHV68 DNA+ cells in each population was determined by a limiting dilution nested PCR assay using the Poisson distribution. In the limiting dilution assays here and in subsequent figures, the dotted line is drawn at 63.2%, and the x-coordinate of the intersection of this line with the sigmoid graph represents the inverse of the frequency of positive events. Frequency of MHV68 DNA+ cells multiplied by 100 is presented as %MHV68+ cells in each B-cell population (**B**). (**C**) Absolute number of MHV68 DNA^+^ cells in each B-cell population (see Materials and Methods for detailed description of how viral reservoir was calculated for this and subsequent figures, as applicable). Each symbol represents data from a single study, with paired observations within the same study connected by a line. *P < 0.05; ns—not significant.

Following intranasal infection of a naïve host, MHV68 undergoes acute lytic replication, which is largely cleared by 10–12 days post-infection, concurrent with the establishment of the splenic latent reservoir that peaks at 16 days post-infection. As the distribution of the latent MHV68 reservoir among B-cell subsets is not stochastic (45), the frequency of MHV68 DNA+ cells was determined in sorted CD11c^+^ and CD11c^neg^ splenic B cells via limiting dilution nested PCR approaches. Using Poisson distribution, a sevenfold increase in the frequency and percent of MHV68 DNA+ cells was observed in CD11c^+^ as compared to CD11c^neg^ splenic B cells (Fig. 1A and B). Furthermore, absolute numbers of MHV68 DNA+ B cells were similar in CD11c^+^ and CD11c^neg^ splenic B-cell populations (Fig. 1C). Thus, infected splenic CD11c^+^ B cells contributed to the overall splenic latent reservoir during the establishment of chronic MHV68 infection.

**Fig 2:**
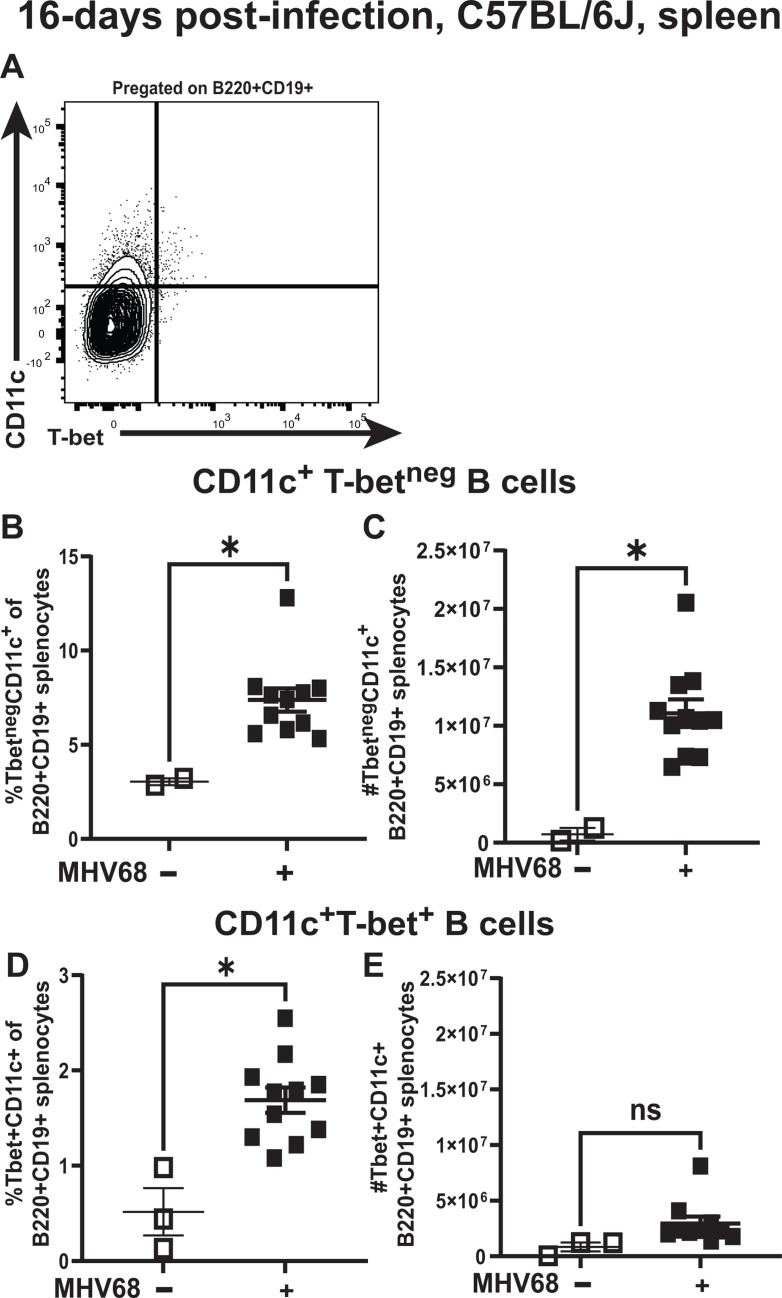
T-bet^+^ and T-bet^neg^ CD11c^+^ splenic B cells increase in proportion and number during the establishment of chronic gammaherpesvirus infection. C57BL/6(J) mice were infected with MHV68 as in Fig. 1 and splenic B cells were analyzed at 16 days post-infection. Splenic B cells were pregated as B220^+^CD19^+^ with CD11c and T-bet expression defined using the gating strategy in **A**. (**B–E**) Proportion and absolute number of splenic B220^+^CD19^+^CD11c^+^T-bet^+^ or B220^+^CD19^+^CD11c^+^T-bet^neg^ B cells are shown. Each symbol represents an individual spleen. *P < 0.05; ns—no statistically significant difference between groups.

Given different published strategies to identify ABCs, including during MHV68 infection, a flow cytometry gating approach described by the Shlomchik group (32) (Fig. 2A) was used to quantify T-bet^+^ and T-bet^neg^ CD11c^+^ B cells at 16 days post-infection, with the latter population not previously assessed. T-bet^+^ and T-bet^neg^ CD11c^+^ splenic B cells increased in proportion, with higher abundance of T-bet^neg^ CD11c^+^ B cells observed at baseline and during chronic infection (Fig. 2B through E).

**Fig 3:**
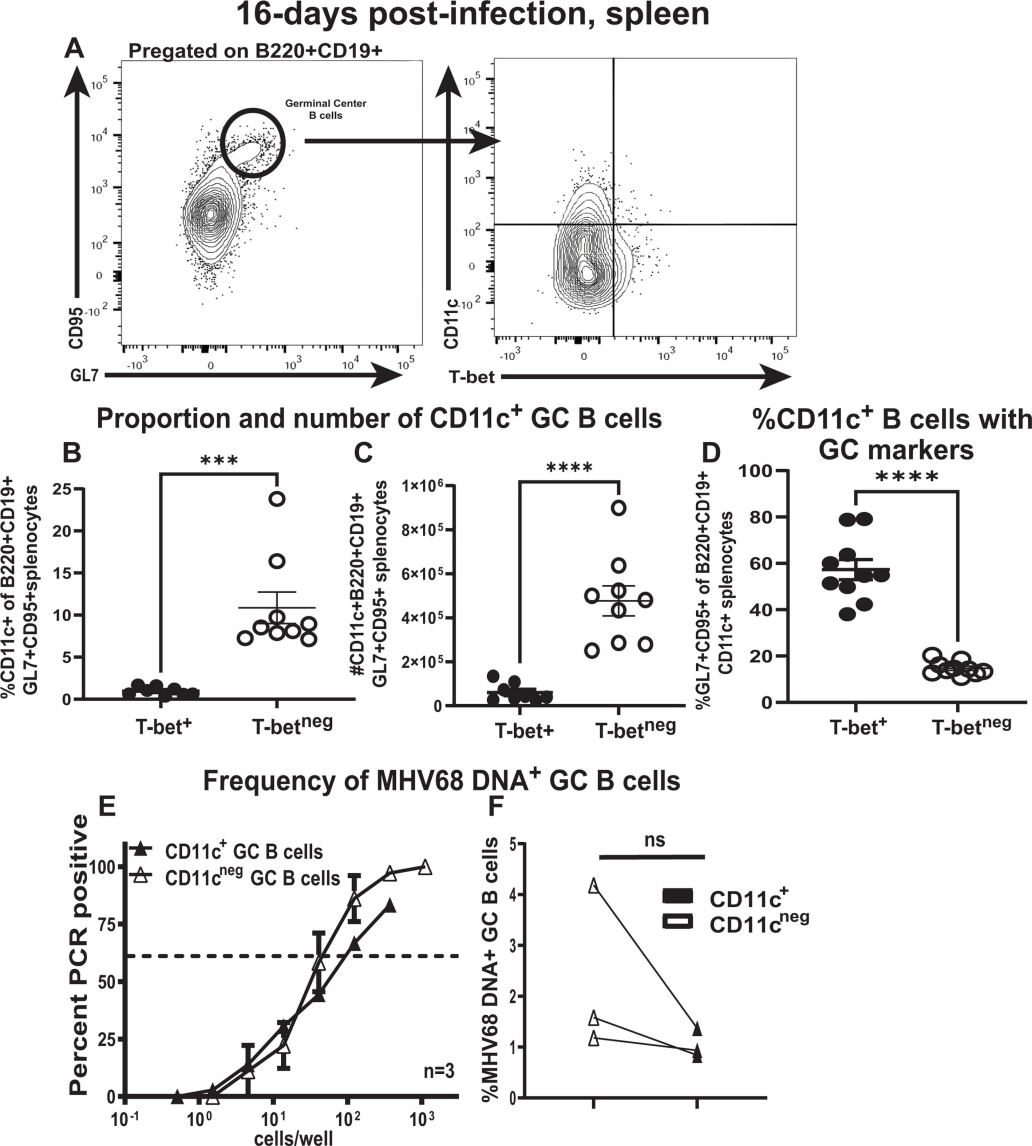
MHV68-driven germinal center differentiation of T-bet^+^ and T-bet^neg^ CD11c^+^ splenic B cells supports the latent viral reservoir. C57BL/6(J) mice were infected with MHV68 as in Fig. 1 and splenic B cells analyzed at 16 days post-infection. (**A**) Flow cytometry gating strategy to quantify expression of CD11c and T-bet by germinal center B cells. (**B, C**). Proportion and absolute number of germinal center B cells represented by T-bet^+^CD11c^+^ or T-bet^neg^CD11c^+^ B cells in the spleens of infected animals. (**D**) Proportion of T-bet^+^CD11c^+^ and T-bet^neg^CD11c^+^ B cells expressing germinal center (GC) markers in spleens from infected animals. (**B–D**) Each symbol represents an individual spleen. (**E, F**) Splenic B cells pooled from infected *Cd19^wt/wt^ Tbx21^fl/fl^* mice were enriched based on CD19 expression and separated into B220^+^CD19^+^GL7^+^CD95^+^CD11c^+^ and B220^+^CD19^+^GL7^+^CD95^+^CD11c^neg^ populations by FACS. The frequency (**E**) of MHV68 DNA+ cells in each population was determined by limiting dilution nested PCR assay using Poisson distribution; data were pooled from three independent experiments. (**F**) %MHV68 DNA+ cells in each germinal center B-cell population. Each symbol represents data from a single study, with paired observations within the same study connected by a line. ***P < 0.001, ****P < 0.0001, ns—no statistically significance differences.

Classically, ABCs are thought to differentiate via the extrafollicular and not the germinal center pathway. In contrast, germinal center B cells support the majority of the latent MHV68 reservoir in the spleen, with a small proportion of the latent reservoir hosted by the marginal zone B cells during the establishment of chronic infection (7, 10). Given the observed enrichment of latent viral reservoir within CD11c^+^ splenic B cells, the expression of germinal center markers was assessed next. Expression of CD11c and T-bet by germinal center B cells was defined using flow cytometry gating strategy in Fig. 3A. Interestingly, 10% and 2% of all germinal center B cells in infected spleens were represented by T-bet^neg^ CD11c^+^ and T-bet^+^ CD11c^+^ B cells, respectively, with correspondingly greater numbers of T-bet^neg^ CD11c^+^ germinal center B cells (Fig. 3B and C). Next, the proportion of T-bet^+^ and T-bet^neg^ CD11c^+^ splenic B cells that also expressed germinal center markers was quantified. A majority (~60%) of T-bet^+^ CD11c^+^ B cells expressed germinal center markers during the establishment of chronic infection, with only a minority (~20%) of T-bet^neg^ CD11c^+^ B cells having a germinal center phenotype (Fig. 3D).

Given that the majority of germinal center B cells were CD11c^neg^ during the establishment of chronic infection, the infection status of CD11c^+^ and CD11c^neg^ germinal center B cells was examined next. Despite the lack of CD11c expression by most germinal center B cells during chronic MHV68 infection, similar frequency and percentage of MHV68 DNA+ B cells were observed in CD11c^+^ and CD11c^neg^ germinal center B cells (Fig. 3E and F). Thus, infected CD11c^+^ B cells, including those driven to undergo the germinal center response, contributed to the MHV68 latent reservoir in the spleen and germinal centers during the establishment of chronic gammaherpesvirus infection.

### B cell-intrinsic T-bet deficiency does not affect expansion and germinal center-based differentiation of T-bet^neg^ CD11c^+^ splenic B cells during the establishment of gammaherpesvirus latency

Effective targeting of CD11c-expressing B cells is not yet possible due to the expression of CD11c by several myeloid cell types and the rapid reconstitution of CD11c+ B cells following targeting of this population in a mixed bone marrow chimera approach. In contrast, several genetic approaches have been validated to target T-bet expression by B cells. Bone marrow chimeras generated by transplanting recipients with 80% B-cell deficient (*µMT-/-*) and 20% *Tbx21^-/-^* (T-bet) bone marrow demonstrated increased copy numbers of MHV68 DNA in the recipients’ spleens at 16 days post-intranasal infection, suggesting a protective role of T-bet-expressing B cells (27). However, the number of gammaherpesvirus episomes per latently infected B cell is variable and dependent on the time post-infection (46). Additionally, the number of viral episomes per cell is dramatically increased in splenocytes that are supporting MHV68 reactivation *in vivo*. Thus, quantification of bulk MHV68 DNA in chronically infected spleens can be significantly influenced by a small number of lytically infected cells and the duration of latent B-cell infection, particularly at 16 days post-viral inoculation.

**Fig 4:**
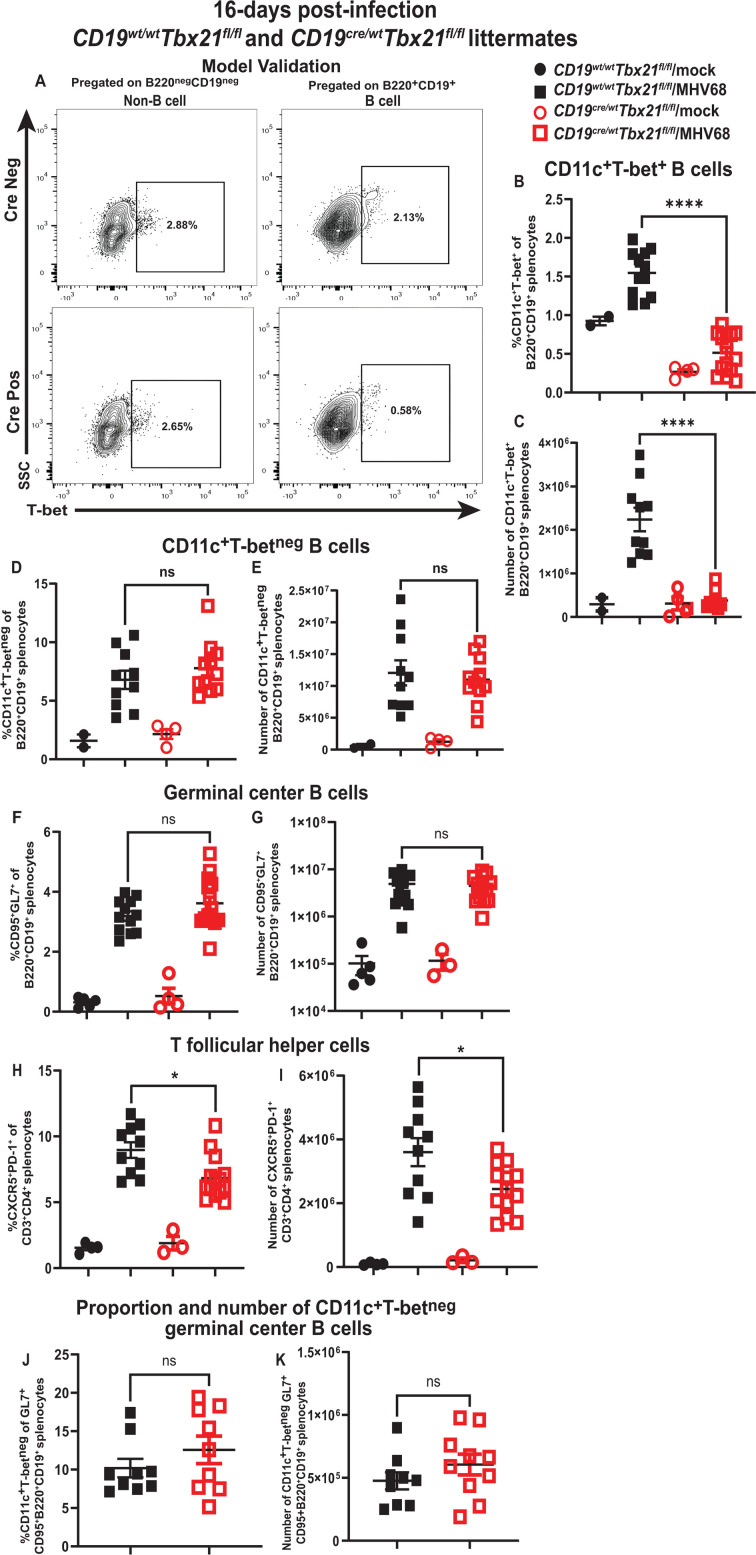
B cell-intrinsic T-bet deficiency does not affect expansion and germinal center-based differentiation of T-bet^neg^ CD11c^+^ splenic B cells during the establishment of chronic gammaherpesvirus infection. *Cd19^Cre/wt^ Tbx21^fl/fl^* and *Cd19^wt/wt^ Tbx21^fl/fl^* littermates were mock-treated or infected as in Fig. 1 and analyzed at 16 days post-infection. (**A**) Representative flow cytometry plot of T-bet expression in splenic B cell and non-B-cell populations of infected mice (*Cd19^Cre/wt^* genotype designated as Cre Pos, *Cd19^wt//wt^* genotype designated as Cre Neg); (**B, C**) The proportion and number of splenic B cells expressing CD11c and T-bet are shown. (**D, E**) The proportion and absolute number of B220^+^CD19^+^CD11c^+^T-bet^neg^ B cells are shown. (**F, G**) The proportion and absolute number of CD95^+^GL7^+^ germinal center B cells are shown. (**H, I**) The proportion and absolute number of T follicular helper cells represented by CD3+CD4+CXCR5+PD-1+ are shown. (**J, K**) The proportion and absolute number of germinal center B cells represented by B220^+^CD19^+^CD11c^+^T-bet^neg^ cells in infected spleens are shown. Each symbol represents an individual spleen. ****P < 0.0001; *P < 0.05; ns—no statistically significant difference between groups.

Given the additional caveats associated with primary gammaherpesvirus infection of aged bone marrow recipients, the role of B-cell-intrinsic T-bet expression during the establishment of chronic MHV68 infection (16 days) was defined using mice with combined CD19-Cre recombinase knock-in (47) and conditional *Tbx21* alleles (48). As expected, validation of this genetic model in the context of chronic MHV68 infection demonstrated a significant decrease in frequency and absolute numbers of MHV68-driven splenic T-bet^+^ CD11c^+^ B cells, whereas other non-B-cell splenic populations retained T-bet expression (Fig. 4A through C).

B cell-specific T-bet deficiency affected neither the magnitude of the MHV68-driven expansion of T-bet^neg^ CD11c^+^ splenic B cells (Fig. 4D and E) nor the overall germinal center B-cell population during the establishment of chronic infection (Fig. 4F and G). CD4+ T follicular helper cells are critically important for MHV68-driven germinal center response (6, 49). Despite the similar magnitude of germinal center B-cell populations in infected groups, the CD4^+^ T follicular helper cell population was decreased in infected mice with B cell-specific T-bet deficiency (Fig. 4H and I). A similar proportion and absolute number of germinal center B cells were represented by the T-bet^neg^ CD11c^+^ B cells, regardless of the *Cd19* genotype (Fig. 4J and K). Thus, B cell-specific T-bet deficiency resulted in attenuated CD4+ T follicular helper cell population but did not affect the MHV68-driven expansion of germinal center B cells and germinal center-based differentiation of T-bet^neg^ CD11c^+^ B cells during the establishment of chronic gammaherpesvirus infection.

### B cell-intrinsic T-bet deficiency leads to a decreased proportion but not absolute number of latently infected CD11c^+^ as compared to CD11c^neg^ germinal center B cells during establishment of chronic infection

**Fig 5:**
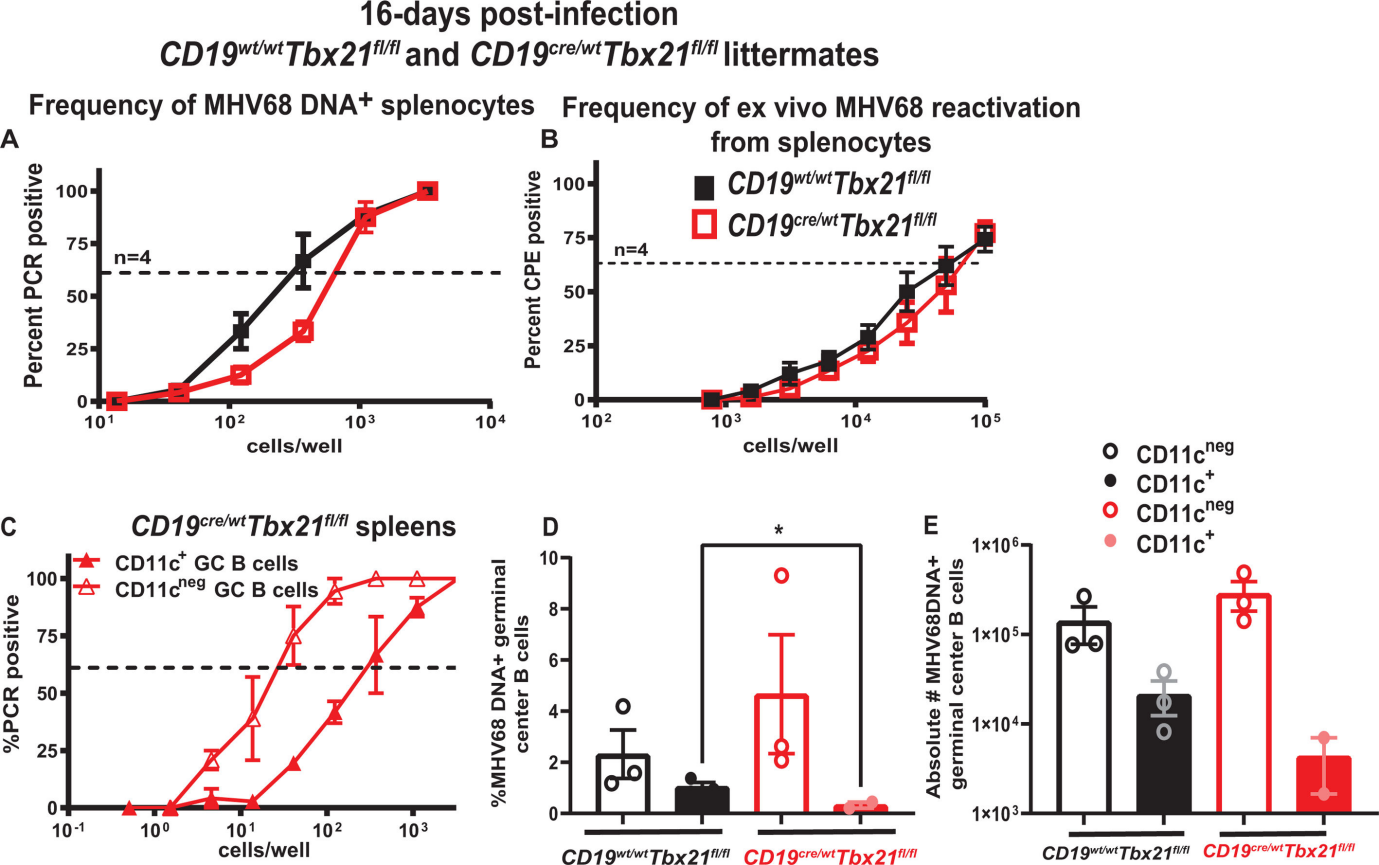
B cell-intrinsic T-bet deficiency leads to a decreased proportion but not absolute number of latently infected CD11c^+^ as compared to CD11c^neg^ germinal center B cells during establishment of chronic infection. *Cd19^Cre/wt^ Tbx21^fl/fl^* and *Cd19^wt/wt^ Tbx21^fl/fl^* littermates were mock-treated or infected as in Fig. 1 and analyzed at 16 days post-infection. (**A, B**) Splenocytes were pooled from mice within each group and subjected to limiting dilution assays to determine the frequency of MHV68 DNA+ cells (**A**) or *ex vivo* reactivation (**B**); data were pooled from four independent experiments. (**C–E**) Splenocytes pooled from infected *Cd19^Cre/wt^ Tbx21^fl/fl^* mice were enriched for CD19^+^ B cells and subsequently separated into B220^+^CD19^+^GL7^+^CD95^+^CD11c^+^ and B220^+^CD19^+^GL7^+^CD95^+^CD11c^neg^ populations by FACS. The frequency (**C**) of MHV68 DNA^+^ cells was measured in each sorted population by limiting dilution nested PCR assay using Poisson distribution; data were pooled from three independent experiments. (**D**) %MHV68 DNA+ cells in each germinal center B-cell population. (**E**) Absolute number of MHV68 DNA+ germinal center B cells in each population. (**E, D**) Each symbol represents data from an independent study. *P < 0.05.

Having observed similar size of germinal center B-cell population in both infected groups (Fig. 4F and G), parameters of MHV68 infection were examined next. A modest decrease in the frequency of MHV68 DNA+ splenocytes, but not *ex vivo* MHV68 reactivation, was observed in mice with B cell-specific T-bet deficiency at 16 days post-infection (Fig. 5A and B). Interestingly, the frequency of MHV68 DNA+ CD11c^+^ as compared to CD11c^neg^ germinal center B cells was decreased in mice with B cell-specific T-bet deficiency (Fig. 5C, compare to Fig. 3E). This decrease was also observed when the inverse of frequency was graphed as percent MHV68 DNA+ germinal center B cells (Fig. 5D). However, when the percentage of MHV68 DNA+ cells was converted to absolute cell numbers, the difference in infected CD11c+ germinal center B cells failed to reach statistical significance (*P* = 0.1, Fig. 5E).

### B cell-specific T-bet deficiency attenuates isotype class switching to IgG_2c_ but not overall IgG titers of self- and MHV68-reactive antibodies during establishment of chronic infection

EBV and MHV68 infections uniquely drive robust differentiation of B cells reactive against self and foreign species antigens, with a peak of self-reactive antibodies observed at 14–16 days post-MHV68 infection (50, 51). In contrast, antiviral antibody titers rise with slower kinetics and do not peak until 30 days post-MHV68 infection. T-bet^Hi^ B cells were important for the generation of protective antibodies against influenza hemagglutinin (25). In contrast, T-bet^+^CD11c^+^ B cells produced autoreactive antibodies in autoimmune mouse models (30, 31). While CD11c^+^ splenic B cells may contribute to MHV68-specific IgG titers *in vivo* (27), the extent to which T-bet^+^ splenic B cells support gammaherpesvirus-driven increase in self-reactive antibody titers is not known.

**Fig 6:**
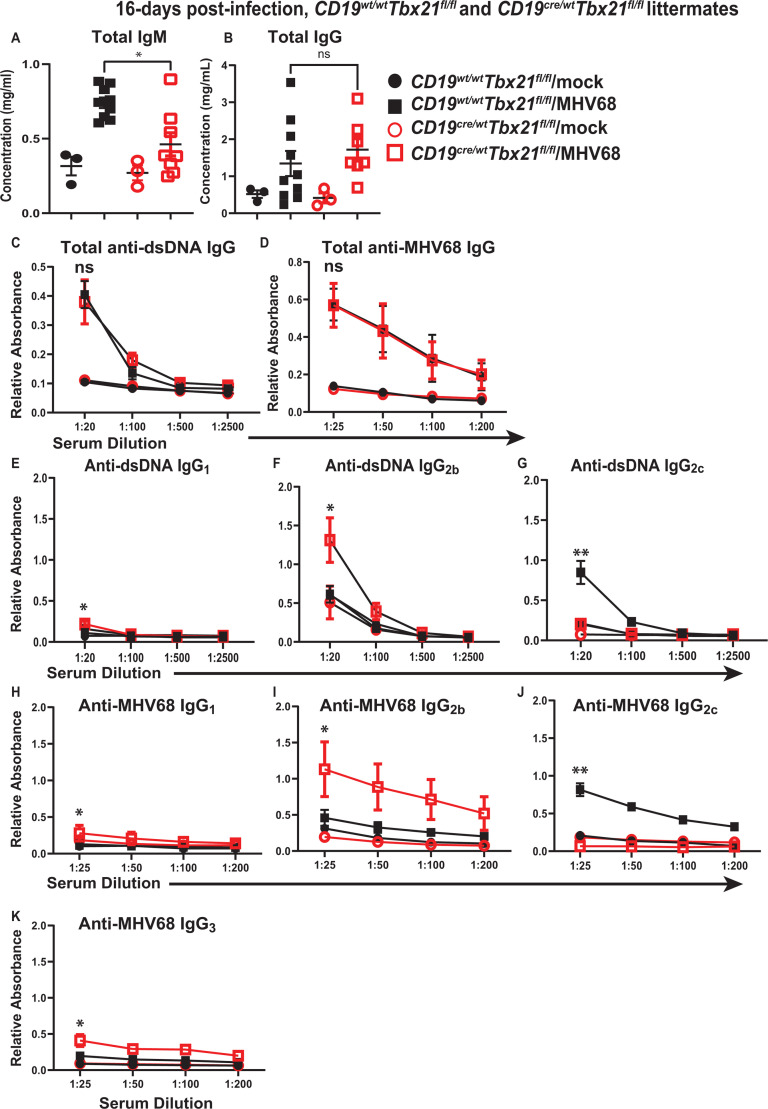
B cell-specific T-bet deficiency attenuates isotype class switching to IgG_2c_ but not overall IgG titers of self- and MHV68-reactive antibodies during establishment of chronic infection. *Cd19^Cre/wt^ Tbx21^fl/fl^* and *Cd19^wt/wt^ Tbx21^fl/fl^* littermates were mock-treated or infected as in Fig. 1, and the serum was analyzed by ELISA at 16 days post-infection for IgM (**A**), IgG (**B**), anti-dsDNA IgG (**C**), and anti-MHV68 IgG (**D**). Anti-dsDNA IgG isotypes (**E–G**) and anti-MHV68 IgG isotypes (**H–K**) were determined. (**A, B**) Each symbol represents an individual mouse. (**C–E**) Data were pooled from three uninfected mice/group and 8–14 infected mice/group. Statistical analyses were limited to infected groups. *P < 0.05; **P < 0.01; ns—no statistically significant difference.

The effect of B cell-specific T-bet deficiency on humoral responses was first defined during the establishment of chronic MHV68 infection. B cell-specific T-bet deficiency resulted in attenuated serum titers of total IgM, but not IgG, in infected mice (Fig. 6A and B, respectively). Correspondingly, B cell-specific T-bet deficiency did not affect self-reactive or MHV68-specific IgG titers, with the former assessed by measuring anti-double-stranded DNA (anti-dsDNA) IgG (Fig. 6C and D).

When isotypes of self-reactive anti-dsDNA IgG antibodies were assessed, IgG_1_ titers were very low to undetectable in all cohorts (Fig. 6E). However, given the large number of mice in infected groups, there was a small yet statistically significant increase in anti-dsDNA IgG1 at the lowest serum dilution of infected mice with T-bet-deficient B cells (Fig. 6E). IgG_2b_ anti-dsDNA titers were increased and, as expected, IgG_2c_ anti-dsDNA titers were decreased in infected mice with B cell-specific T-bet deficiency (Fig. 6F and G). There was no detectable increase in anti-dsDNA IgG_3_ in any group (data not shown). Similar changes in MHV68-specific IgG isotype titers (decrease in IgG_2c_ and increase in IgG_2b_) were observed in mice with B cell-specific T-bet deficiency (Fig. 6H through J), with a very small, but statistically significant increase in IgG_3_ MHV68-specific antibodies in *Cd19^cre/wt^Tbx21^fl/fl^* mice (Fig. 6K). Thus, B cell-intrinsic T-bet expression was important for the generation of MHV68-driven self-reactive and MHV68-specific IgG_2c_ antibodies. In addition, loss of B cell-intrinsic T-bet expression resulted in a compensatory increase in IgG_2b_ titers, with no effect on the overall class-switched antiviral or self-reactive IgG titers at 16 days post-infection.

### B cell-intrinsic T-bet expression attenuates the MHV68-driven germinal center response, differentiation of self-reactive B cells, and latent viral reservoir during long-term infection

**Fig 7:**
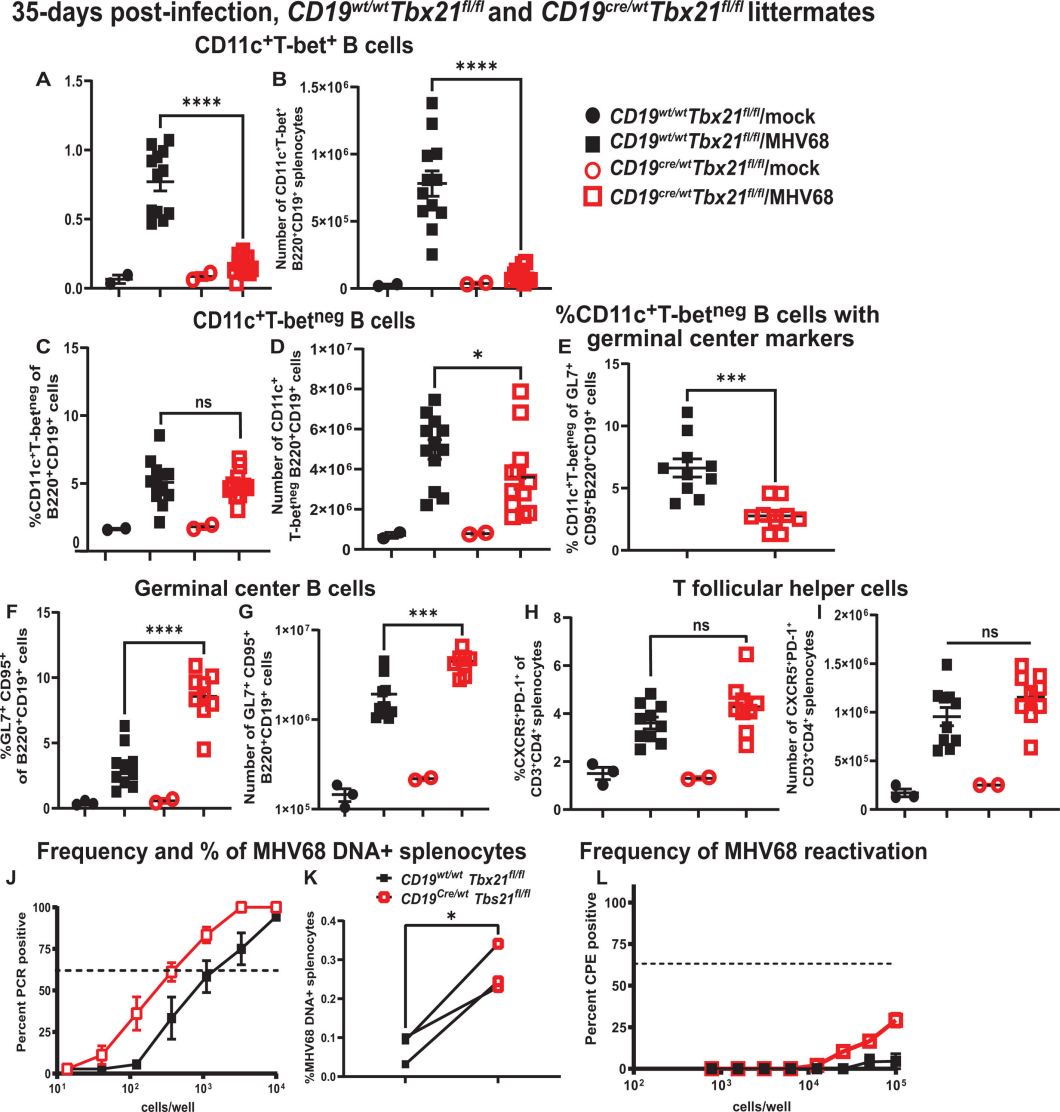
B cell-intrinsic T-bet expression attenuates the MHV68-driven germinal center response and the latent viral reservoir during long-term infection. *Cd19^Cre/wt^ Tbx21^fl/fl^* and *Cd19^wt/wt^ Tbx21^fl/fl^* littermates were mock-treated or infected as in Fig. 1 and splenocytes analyzed at 35 days post-infection. (**A, B**) The proportion and absolute number of B220^+^CD19^+^CD11c^+^T-bet^+^ B cells are shown. (**C, D**) The proportion and absolute number of B220^+^CD19^+^CD11c^+^T-bet^neg^ B cells are shown. (**E**) The proportion of germinal center B cells represented by B220^+^CD19^+^CD11c^+^T-bet^neg^ cells in infected spleens is shown. (**F, G**) The proportion and absolute number of CD95^+^GL7^+^ germinal center B cells are shown. (**H, I**) The proportion and absolute number of CD3^+^CD4^+^CXCR5^+^PD-1^+^ T follicular helper cells are shown. (**A–I**) Each symbol represents an individual spleen. (**J–L**) Splenocytes were pooled within each group and subjected to limiting dilution PCR (**J**) or limiting dilution *ex vivo* reactivation assay (**L**) to determine the frequency of MHV68 DNA^+^ cells (**J**) or the frequency of *ex vivo* reactivation (**L**). (**J, L**) Data were pooled from three independent experiments. (**K**) Data represent % of MHV68 DNA+ splenocytes. Each symbol represents data from a single study, with paired observations within the same study connected by a line.*P < 0.05; **P < 0.01; ***P < 0.001, ****P < 0.0001; ns—no statistically significant difference.

Following its peak at 16 days post-infection, the splenic latent MHV68 reservoir contracts and stabilizes by 35 days post-infection, along with a significant decrease in MHV68-driven differentiation of self-reactive B cells (51). To determine the extent to which B cell-specific T-bet expression is required for the maintenance of CD11c^+^ B cells during long-term infection, splenic T-bet^+^ and T-bet^neg^ CD11c^+^ B cells were measured at 35 days post-infection. While splenic T-bet^+^ CD11c^+^ B cells remained elevated in control long-term infected mice, a significant depletion of T-bet^+^ CD11c^+^ B cells continued to be observed in long-term infected mice with a B cell-specific T-bet deficiency (Fig. 7A and B). In contrast to that observed at 16 days post-infection, B cell-specific T-bet deficiency resulted in decreased absolute numbers, but not frequency, of T-bet^neg^ CD11c^+^ splenic B cells (Fig. 7C and D). Consistent with decreased abundance of T-bet^neg^ CD11c^+^ splenic B cells in long-term infected mice with B cell-specific T-bet deficiency, decreased proportion of germinal center B cells was represented by T-bet^neg^ CD11c^+^ cells (twofold, Fig. 7E). Interestingly, and in contrast to that observed at 16 days post-infection, B cell-specific T-bet deficiency led to a sustained increase in the frequency and absolute number of germinal center B cells in long-term infected mice (~2.7 fold, Fig. 7F and G), while the frequency and absolute number of T follicular helper cells were similar in both infected groups (Fig. 7H and I). Consistent with increased numbers of germinal center B cells, an increase in the splenic latent viral reservoir was observed in mice with B cell-specific T-bet deficiency (3.6-fold, Fig. 7J and K). MHV68 reactivation from splenocytes is largely suppressed in long-term infected mice following intranasal infection. However, a small increase in the frequency of MHV68 reactivation was observed in splenocytes of long-term infected mice with B cell-specific T-bet deficiency; this increase could not be quantified or statistically analyzed due to the very low levels of reactivation (Fig. 7L).

**Fig 8:**
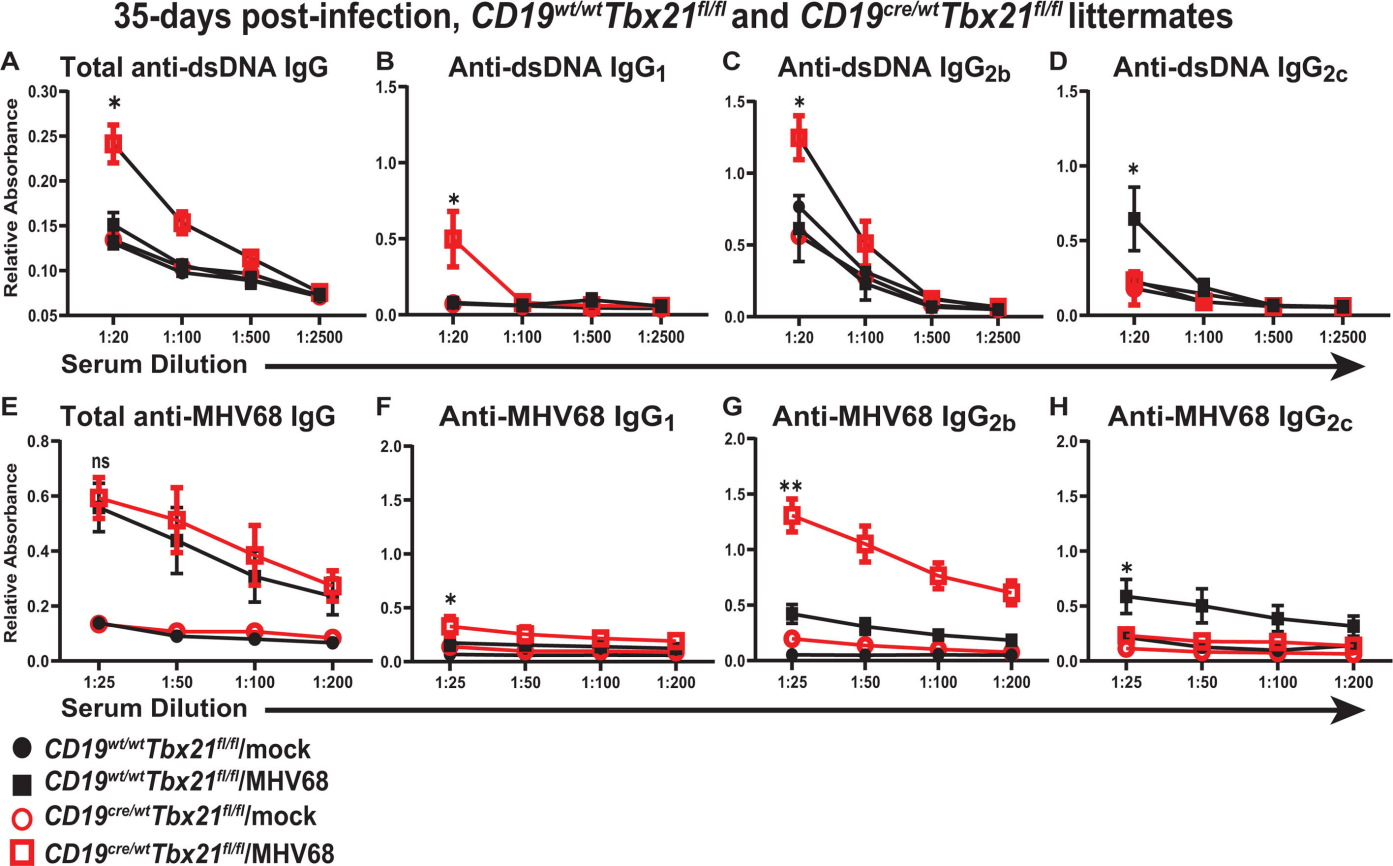
B cell-intrinsic T-bet expression attenuates the MHV68-driven differentiation of self-reactive B cells during long-term infection. *Cd19^Cre/wt^ Tbx21^fl/fl^* and *Cd19^wt/wt^ Tbx21^fl/fl^* littermates were mock-treated or infected as in Fig. 1, and the sera were analyzed at 35 days post-infection. Total serum IgG and IgG isotype titers were determined for anti-dsDNA antibodies (**A–D**) and anti-MHV68 antibodies (**E–H**). *P < 0.05; **P < 0.01; ns—no significant difference between groups.

As expected, the titers of dsDNA-reactive total IgG returned to near-baseline levels in long-term infected control (*Cd19^wt/wt^Tbx21^fl/fl^*) mice (Fig. 8A). However, total anti-dsDNA IgG titers remained elevated in long-term infected *Cd19^cre/wt^Tbx21^fl/fl^* mice (Fig. 8A), including those of the IgG_1_ and IgG_2b_ isotypes, with expected decrease in the IgG_2c_ anti-dsDNA titers (Fig. 8B through D). In contrast, total IgG titers of anti-MHV68 antibodies remained similar in long-term infected mice regardless of the *Cd19* genotype (Fig. 8E). Anti-MHV68 IgG antibodies were primarily represented by IgG_2c_ in long-term infected control mice, with compensatory increases in MHV68-specific IgG_2b_, and to a lesser extent, IgG_1_ in mice with B cell-specific T-bet deficiency (Fig. 8F through H). In summary, B cell-specific T-bet expression attenuated the MHV68-driven germinal center response, differentiation of self-reactive B cells, and the splenic latent reservoir during long-term infection.

## DISCUSSION

Gammaherpesviruses are B cell-tropic viruses that infect and manipulate B cells to amplify the latent viral reservoir in germinal centers that are also thought to seed viral lymphomagenesis (5). ABCs represent a heterogeneous collection of B cells in humans and animals, with many ABC subsets expressing CD11c and/or T-bet. Using a highly tractable MHV68 gammaherpesvirus infection model, this study is the first to demonstrate enrichment of the latent viral reservoir in splenic CD11c^+^ B cells during establishment of chronic gammaherpesvirus infection in a natural host. We showed that T-bet^+^ and T-bet^neg^ CD11c^+^ B cells expanded during chronic gammaherpesvirus infection, acquired expression of germinal center markers, and that CD11c^+^ B cells supported latent viral reservoir in germinal centers. While B cell-intrinsic T-bet expression had minimal effects on viral and host parameters during the establishment of chronic gammaherpesvirus infection, it facilitated optimal control of the long-term splenic latent reservoir, gammaherpesvirus-driven germinal center response, and differentiation of self-reactive B cells.

### CD11c^+^ B cell dynamics during chronic gammaherpesvirus infection

This study demonstrated that during chronic gammaherpesvirus infection, T-bet^+^ and T-bet^neg^ CD11c^+^ B cells expanded and expressed germinal center markers. This germinal center-based differentiation is physiologically relevant, as a majority of MHV68-infected cells localize to the germinal centers at 16 days post-infection (7). Here, we showed enrichment of latent infection in CD11c^+^ B cells. Although splenic T-bet^neg^ CD11c^+^ B cells were more abundant as compared to T-bet^+^ CD11c^+^ B cells, only ~20% of the former expressed germinal center B-cell makers, whereas ~60% of the less abundant T-bet^+^CD11c^+^ B cells expressed the germinal center markers GL7 and CD95 during the establishment of chronic infection. This observation suggests that either T-bet^+^ CD11c^+^ B cells preferentially enter the germinal center independent of their infection status or that MHV68-infected CD11c^+^ B cells are more likely to induce T-bet expression and undergo germinal center differentiation, two scenarios that require further investigation.

Classically, ABCs were thought to undergo extrafollicular differentiation, with their entry into the germinal center questioned (22). However, the physiological role of germinal center-based differentiation of T-bet^+^ B cells demonstrated in this study is congruent with other recently published studies. In a mouse model of malaria infection, germinal center B cells increased T-bet expression, leading to increased somatic hypermutation and antibody avidity against the parasite (52). While we showed that most CD11c^+^ germinal center B cells were T-bet^neg^ at 16 days post-MHV68 infection, T-bet^+^ B cells dominated the germinal center during influenza infection and were responsible for the generation of protective anti-hemagglutinin antibody (25, 26). In contrast, fate mapping of T-bet^+^CD11c^+^ during acute LCMV infection showed their development to be largely germinal center-independent (53).

This study demonstrates that the numbers of latently infected B cells were similar in CD11c^+^ and CD11c^neg^ splenic B-cell populations, despite CD11c^+^ B cells representing a small proportion of the overall splenic B-cell population. It is possible that MHV68 preferentially infects and/or maintains latency in CD11c^+^ B cells. In an alternative non-exclusive scenario, MHV68 infection of a naïve CD11c^neg^ splenic B cell promotes its differentiation into CD11c^+^ ABC. This alternative scenario is plausible given the well-established roles of TLR7 and TLR9 in ABC differentiation (22), the same TLRs that are engaged by gammaherpesvirus infection (54). Additional studies are needed to discern between these two possible scenarios and evaluate the differentiation and infection status of T-bet^+^ B cells during MHV68 chronic infection.

Enrichment of latent gammaherpesvirus infection within CD11c^+^ B cells remains to be formally demonstrated during chronic EBV or KSHV infection. However, *de novo* EBV infection of cultured human peripheral B cells led to a robust increase in CD11c^+^ B cells by 5 days post-infection (55). Additionally, spontaneous *in vitro* transformation of peripheral B cells by endogenous EBV was only observed in healthy human donors over 45 years of age (56), consistent with age-dependent increases in ABCs. Unlike that observed in healthy donors, spontaneous *in vitro* transformation by endogenous EBV was evident in young donors with active multiple sclerosis, with CD11c expression by transformed B cells further increased in multiple sclerosis as compared to healthy donor-derived B cell lines (56).

### Control of chronic gammaherpesvirus infection by T-bet^+^ B cells

While B-cell-specific T-bet deficiency had minimal viral and host effects during the establishment of MHV68 chronic infection, it was required for optimal control of the long-term latent reservoir (with minimal effect on viral reactivation) and MHV68-driven pathogenic germinal center responses. Our observations are different from those reported by the Horwitz group that demonstrated similar viral DNA copy numbers but significantly increased splenic MHV68 reactivation at 35 days post-infection in mice with B-cell-specific T-bet deficiency (38, 39). The published studies from the Horwitz group exclusively used the intraperitoneal route of MHV68 inoculation, whereas the current study utilized intranasal MHV68 infection. Herpesviruses, including gammaherpesviruses, transmit via direct contact with mucosal surfaces. In contrast, intraperitoneal inoculation of gammaherpesviruses in humans is likely limited to iatrogenic infection. We and others reported profound changes in the viral and host parameters of MHV68 infection imposed by intranasal vs. intraperitoneal viral inoculation, including cell types supporting latent MHV68 reservoir and parameters of reactivation (40–44). Additionally, publications from the Horwitz group used a real-time PCR-based assay to measure MHV68 genomes in bulk splenic DNA normalized to total splenocyte numbers. In contrast, limiting dilution nested PCR assays were used in the present study to quantify the frequency of MHV68 DNA+ splenocytes. The choice of assay for the current study was guided by the observation that the number of gammaherpesvirus episomes per latently infected B cell is variable and depends on the time post-infection (46). Furthermore, viral reactivation is expected to produce a dramatic increase in MHV68 DNA in the few splenocytes supporting lytic replication. Thus, the use of limiting dilution approaches to quantify the frequency of splenocytes harboring >5 copies of MHV68 genome (sensitivity of the nested PCR assay, data not shown) eliminated the caveats associated with the bulk measurements of MHV68 DNA during chronic infection, as outlined above.

B cell-specific T-bet deficiency had no effect on the overall antiviral IgG titers at any examined time post-MHV68 infection, although the isotypes of antiviral antibodies were impacted. While antiviral antibodies play a critical role in many viral infections, MHV68 infection is well controlled in a host where B cells can only produce hen egg lysozyme-specific antibodies (57). Thus, the observed increase in the latent MHV68 reservoir is unlikely to be driven by an altered antiviral humoral response. In contrast, B cell-specific T-bet deficiency led to sustained increase in self-reactive antibody titers, as measured by dsDNA IgG, at 35 days post-infection. The observed increase in self-reactive antibody titers was an intriguing observation as gammaherpesvirus infection drives a robust, yet transient expression of self- and foreign species-specific antibodies that wanes by ~28 days post-MHV68 infection (50, 51, 58). Like that of the antiviral IgG antibody titers, dsDNA IgG isotype class-switching was affected in mice with B cell-specific T-bet deficiency. B cell-specific T-bet expression is required for the IgG_2a/2c_ class-switching; thus, the observed attenuation of antiviral and dsDNA IgG_2c_ and compensatory increase in IgG_2b_ titers was anticipated (59).

Given the preferential latent infection of germinal center B cells, an increased germinal center response is a likely explanation for the increase in latent MHV68 reservoir observed in long-term infected mice with B cell-specific T-bet deficiency. MHV68-driven B-cell differentiation is dependent on T follicular helper cells and MHC-II expression by B cells (6, 50). Given the well-established role of T-bet^+^ B cells as antigen-presenting cells (31, 60, 61), it is tempting to speculate that loss of this B-cell population leads to altered differentiation of T follicular helper cells. Intriguingly, the T follicular helper cell population was decreased in mice with B-cell-specific T-bet deficiency at 16 days post-infection, despite similar levels of germinal center B cells in both infected groups (Fig. 4). Furthermore, despite increased germinal center B cells in long-term infected mice with B cell-specific T-bet deficiency, T follicular helper population was not altered by the *Cd19* genotype (Fig. 7), despite higher titers of self-reactive IgG antibodies (Fig. 8). These observations suggest that B cell-intrinsic T-bet expression has quantitative and qualitative effects on the T cell-dependent gammaherpesvirus-driven germinal center response, with the mechanisms to be defined in future studies. Future studies are also needed to define the extent to which B cell-intrinsic T-bet expression controls gammaherpesvirus pathogenesis, including lymphomagenesis and autoimmune disease.

## MATERIALS AND METHODS

### Animal studies

C57BL/6J mice were purchased from The Jackson Laboratory. *Cd19^Cre/wt^ Tbx21^fl/fl^* and *Cd19^wt/wt^ Tbx21^fl/fl^* mice were provided by Dr. Elizabeth Leadbetter (48). Mice were bred and housed in a specific pathogen-free facility at MCW. At 6–7 weeks of age, mice were intranasally infected under light anesthesia with 10,000 PFU of MHV68 diluted in sterile, serum-free Dulbecco’s modified Eagle’s medium immediately prior to infection. MHV68 viral stock was prepared and titered using the 3T12 fibroblast cell line.

### Flow cytometry

Single-cell suspensions of splenocytes were prepared in FACS buffer (phosphate-buffered saline [PBS], 2% fetal bovine serum), and 2 × 10^6^ nucleated cells were surface-stained with optimal antibody concentrations at 4°C. For intracellular staining, cells were fixed and permeabilized using FOXP3 Fix/Perm Buffer Set (cat: 421403; BioLegend [San Diego, CA]) according to the manufacturer’s instructions, and cells were stained for 1 h with optimal antibody concentrations. Data acquisition was performed on BD FACSCelesta (BD Biosciences, San Jose, CA) and Aurora Cytek cytometers (Cytek Biosciences, Fremont, CA). The following antibodies were purchased from BioLegend (San Diego, CA) for use in this study: B220-PECy7 (cat. 103222), CD19-PB (cat. 115523), GL7-FITC (cat. 144604), CD95-APC (cat. 152604), T-bet-PE, CD11c-BV421, and CD19-APCy7.

### ELISAs

Total IgG, total IgM, viral-specific, and anti-dsDNA ELISAs were performed as previously described (62). In brief, Nunc MaxiSorp Plates were coated with anti-IgG (heavy + light), UV-inactivated MHV68, or *E. coli* dsDNA overnight at 4°C. Serum dilutions were added to coated plates, and bound antibodies were detected using horseradish peroxidase (HRP)-conjugated goat anti-mouse total IgG, IgM, IgG1, IgG2b, IgG2c, and IgG3 (heavy plus light chain) (Jackson ImmunoResearch, West Grove, PA) using 3,3′,5,5′-tetramethylbenzidine substrate (Life Technologies, Gaithersburg, MD). HRP activity stopped by the addition of 1 N HCl, and absorbance was read.

### Cell sorting

Splenocytes from each experimental group (3–5 mice/group) were pooled at 16 days post-infection. B cells were enriched using EasySep Mouse CD19 Positive Selection Kit (Stemcell Technologies, Vancouver, CA) according to the manufacturer’s instructions. In brief, a concentration of 1 × 10^8^ splenocytes/mL of sorting media (PBS, 2% FBS, 1mM EDTA) was prepared. 50 µL of selection cocktail was added per 1 mL of cell suspension, followed by a 5-min incubation at room temperature. After incubation, 75 µL of RapidSpheres was added per 1mL of cell suspension and incubated at room temperature for 3 min. 1.5 mL of sorting media was then added before placement into the EasySep magnet for 3 min. The cells mobilized to the tube wall by the magnet (B cells) were resuspended in 1 mL of fresh sorting media and surface-stained as described. Enriched B cells were sorted by surface marker expression on BD FACSAria III Cell Sorter (BD Biosciences, San Jose, CA) prior to LDPCR analysis.

### Limiting dilution assays

The frequency of MHV68 DNA-positive cells was determined as previously described (63). Specifically, splenocytes were pooled from all mice in each experimental group (3–5 mice/group) and six, 3-fold serial dilutions were subjected to nested PCRs (12 replicates/dilution) using primers against the viral genome. Frequency of MHV68 DNA+ cells was determined using Poisson distribution, and the inverse of frequency was presented as %MHV68+ cells in analyzed B-cell populations. The %MHV68+ cell values were used to determine the absolute number of MHV68 DNA^+^ cells in B-cell populations of interest. To determine the frequency of *ex vivo* MHV68 reactivation, splenocytes and peritoneal cells were pooled from all mice within each experimental group (3–5 mice/group), and eight 2-fold serial dilutions of cell suspensions from each group were plated onto a monolayer of MEFs at 24 replicates per dilution. To control for preformed virus four, 2-fold serial dilutions of mechanically disrupted splenic and peritoneal cells were plated on MEFs. Viral reactivation, as indicated by cytopathic clearing of MEFs, was assessed on day 21 of culture.

### Calculations of viral reservoir and MHV68+ cell populations

The frequency of positive events (infected cells) in limiting dilution assays was derived using the Poisson distribution. Frequency was multiplied by 100 to obtain the percent positive cells in an examined population. The absolute number of infected cells was determined by multiplying frequency by the average number of cells in the examined population per mouse (i.e., splenocytes, B cells, or germinal center B cells, as indicated in the figures).

### Statistical analyses

Statistical analyses were performed using Student’s t-test (Prism, GraphPad Software, Inc.).
